# Plant Lectins Targeting *O*-Glycans at the Cell Surface as Tools for Cancer Diagnosis, Prognosis and Therapy

**DOI:** 10.3390/ijms18061232

**Published:** 2017-06-09

**Authors:** Guillaume Poiroux, Annick Barre, Els J. M. van Damme, Hervé Benoist, Pierre Rougé

**Affiliations:** 1 Institut National de la Santé et de la Recherche Médicale, Unité Mixte de Recherche, Centre de Recherche en Cancérologie de Toulouse, 31037 Toulouse, France; guillaume.poiroux@inserm.fr; 2 Unité Mixte de Recherche, 152 PharmaDev, Institut de Recherche et Développement, Faculté de Pharmacie, 35 Chemin des Maraîchers Université Paul Sabatier, 31062 Toulouse, France; annick.barre@univ-tlse3.fr (A.B.); herve.benoist@ird.fr (H.B.); 3 Department of Molecular Biotechnology, Faculty of Bioscience Engineering, Ghent University, Coupure links 653, B-9000 Ghent, Belgium; ElsJM.VanDamme@UGent.be

**Keywords:** lectin, *O*-glycosylation, Tn antigen, T antigen, Morniga G, peanut lectin, cancer, diagnosis, prognosis, photodynamic therapy

## Abstract

Aberrant *O*-glycans expressed at the surface of cancer cells consist of membrane-tethered glycoproteins (T and Tn antigens) and glycolipids (Lewis a, Lewis x and Forssman antigens). All of these *O*-glycans have been identified as glyco-markers of interest for the diagnosis and the prognosis of cancer diseases. These epitopes are specifically detected using T/Tn-specific lectins isolated from various plants such as jacalin from *Artocarpus integrifola*, and fungi such as the *Agaricus bisporus* lectin. These lectins accommodate T/Tn antigens at the monosaccharide-binding site; residues located in the surrounding extended binding-site of the lectins often participate in the binding of more extended epitopes. Depending on the shape and size of the extended carbohydrate-binding site, their fine sugar-binding specificity towards complex *O*-glycans readily differs from one lectin to another, resulting in a great diversity in their sugar-recognition capacity. T/Tn-specific lectins have been extensively used for the histochemical detection of cancer cells in biopsies and for the follow up of the cancer progression and evolution. T/Tn-specific lectins also induce a caspase-dependent apoptosis in cancer cells, often associated with a more or less severe inhibition of proliferation. Moreover, they provide another potential source of molecules adapted to the building of photosensitizer-conjugates allowing a specific targeting to cancer cells, for the photodynamic treatment of tumors.

## 1. Introduction

The malignant transformation is accompanied by profound alterations in both the *N*- and *O*-glycosylation processes in healthy cells [1,2,3]. In cancer cells, the aberrant *O*-glycans expressed at the cancer cell surface occur as saccharide components of membrane-bound *N*-acetyl galactosamine (*O*-GalNAc) glycoproteins (T and Tn antigen) and glycolipids (Lewis a and Lewis x). The occasional sialylation of the ultimate sugar of the glycan chain introduces an additional diversity in the *O*-glycan repertoire expressed by cancer cells [4,5,6,7,8,9,10,11,12]. In addition, mucin, a heavily *O*-GalNAc glycosylated protein, is overexpressed and subsequently secreted by cancer cells, essentially at the last stages of the malignant progression [13,14]. All of these aberrant *O*-glycans may serve as potential targets to improve the diagnosis and the treatment of tumors, provided the molecular probes are available for their specific recognition [15,16,17]. In this respect, monoclonal antibodies that specifically recognize both the sialylated and non-sialylated Tn and T antigens have been widely used to detect malignant cells [18,19,20,21,22]. Lectins and, especially, plant and fungal lectins that display a T/Tn-specificity, consist of another source of potential molecular probes available for the specific recognition of tumor cell *O*-glycans [23]. During the last decade, the list of T/Tn-specific lectins isolated and characterized from plants and fungi, has increased tremendously, making new lectins available as potential molecular probes for the recognition of cancer cells (see Table 1). In parallel, new insights have been obtained on the immunotoxicity of plant lectins toward cancer cells and their role in the reinforcement of the innate (anti-cancer) immunity (see [24] for a review), which assign plant and fungal lectins as valuable tools for the diagnosis and treatment of cancer. Here, we present an updated review on the potential use of plant and fungal lectins as probes for both the diagnosis, the prognosis, and the treatment of cancer.

## 2. Altered *O*-Glycan Patterns Expressed by Cancer Cells

**Figure 1:**
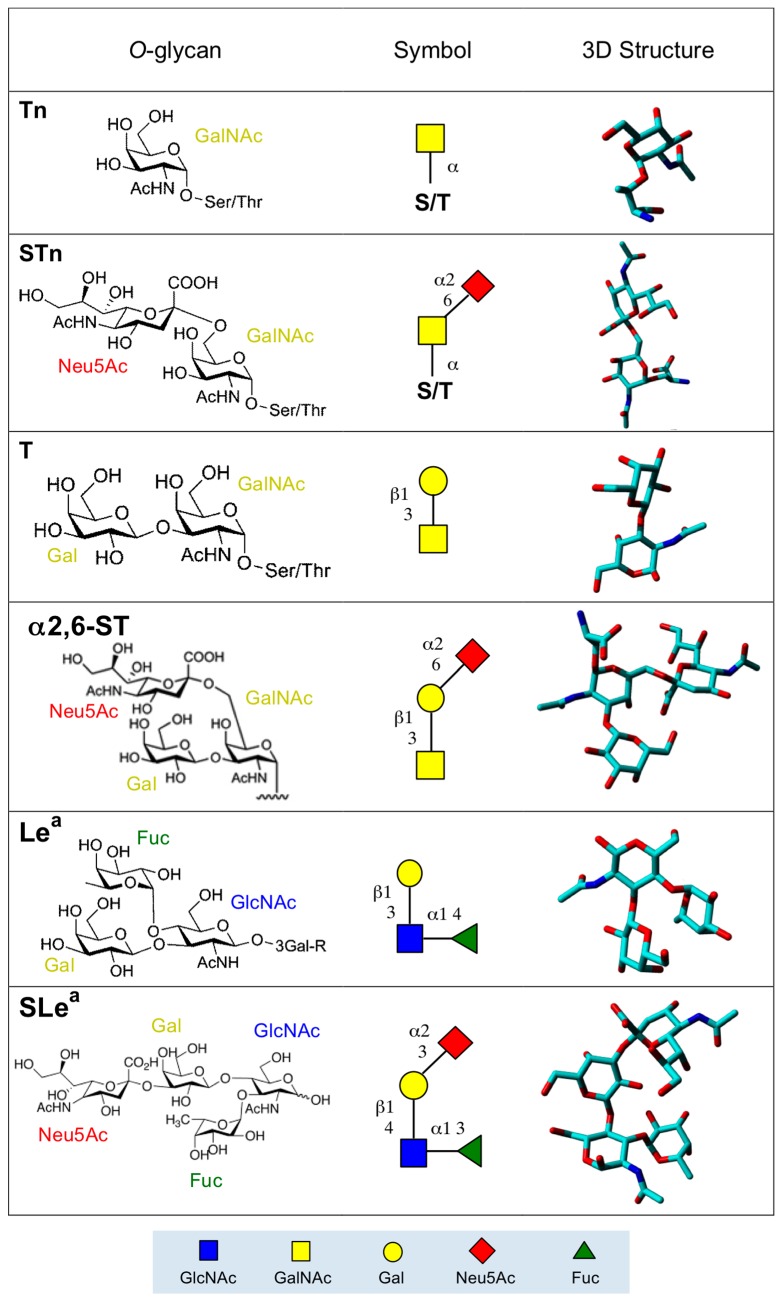
Molecular structure of the *O*-glycans expressed on the cancer cell surface. T antigen also occurs as a component of the soluble mucin excreted by both healthy and cancer cells. GlcNAc, *N*-acetyl d-glucosamine; GalNAc, *N*-acetyl d-galactosamine; Gal, d-galactose; Neu5Ac, sialic acid; Fuc, l-fucose.

**Figure 2:**
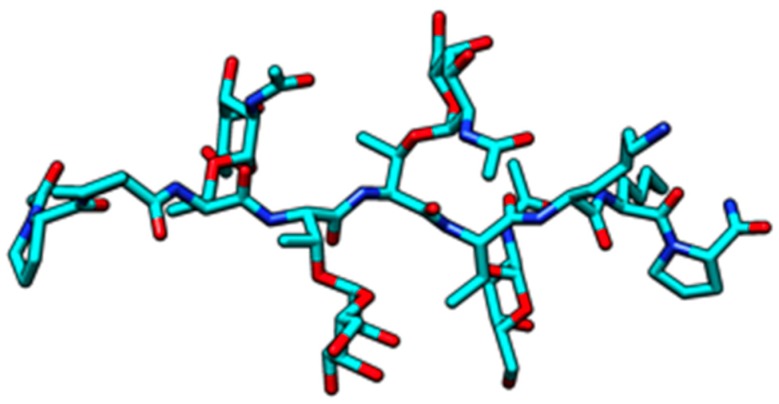
Cartoon showing the clustering of Tn antigens along the peptide chain of the tetra-*O*-GalNAc glycosylated mucin sequence of the human α-dystroglycan mucin domain peptide (residues 419-PPTTTTKKP-427) (PDB code 2MK7; Borgert A, Foley L, Live D). Cartoon drawn with Chimera [27].

Alterations to surface properties of cancer cells account for their aptitude to aggregate and, thus, improve the invasive and metastatic capacity of many tumors. Changes of the surface properties of cancer cells essentially depend on the overexpression of aberrant *O*- and *N*-glycans, which occur as membrane-associated glycoproteins and glycolipids exposed at the cell surface [1]. Due to their high occurrence in most cancer cells, alterations of the *O*-glycosylation have been deeply investigated since the characterization of the so-called T antigen (Thomsen–Friedenreich) and Tn antigen [4]. The most frequent aberrant *O*-glycans expressed at the surface of cancer cells consist of Tn antigen (α1→Ser/Thr), T antigen (Galβ1→3GalNAcα1→Ser/Thr), Lewis a (Galβ1→3[Fucα1→4]GlcNAcβ1→R) and Lewis x (Galβ1→4[Fucα1→3]GlcNAcβ1→R) antigens, and an oncofetal glycotope, the Forssman pentasaccharide antigen (GalNAcα1→3GalNAcβ1→3Galα1→4Galβ1→4Glc) [1,2,3] (Figure 1). All of these glycotopes also exist as sialylated forms, with Neu5Acα2→3-linked to the ultimate Gal (T antigen, Lewis a and Lewis x antigen) or GalNAc residue (Tn antigen) (Figure 1). Except for the Forssman antigen, other antigens relate to the blood group antigen precursors, MN antigens for Tn and T antigen, ABH antigens for Lewis a and Lewis x antigen. Finally, malignancy is often associated with the overproduction of secreted and membrane-tethered mucins [25], glycoproteins which consist of tandemly repeated Tn antigen units (PDB code 2MK7) (Figure 2).

All of these membrane-associated *O*-glycans aberrantly expressed in cancer cells represent cancer glyco-markers that may be recognized using specific monoclonal antibodies or T/Tn-specific lectins as probes. In this respect, plant and fungal lectins displaying a functional T/Tn-specificity mimic the galectins, which innately occur in humans and other mammal organisms as endogenous recognition factors for the aberrant *O*-glycans exposed at the tumor cell surface [26]. However, beyond this apparently functional similarity, exogenous plant lectins and endogenous galectins readily differ from the monoclonal antibodies used as *O*-glycan probes by the topography and the molecular mechanism occurring at their *O*-glycan-binding sites.

## 3. Plant Lectins Specific for T and Tn Antigens

Thus far, up to forty-six lectins isolated from different families of plants and fungi have been characterized as T/Tn-specific lectins (Table 1). Plant lectins have originated from species belonging to a few predominant families such as Fabaceae (BPA from *Bauhinia purpurea*, Gs I-A_4_ from *Griffonia simplicifolia*, PNA from peanut, SBA from soybean, VVA B4 from *Vicia villosa*, WBL from the winged bean *Psophocarpus tetragonolobus*, and WFA from *Wisteria floribunda*), Caprifoliaceae (SNA-I, SNA-II and SNA-IV from *Sambucus nigra*), Lamiaceae (SSA from *Salvia sclarea*, SHA from *S. hominum*, and SbA from *S. bogotensis*), Euphorbiaceae (ricin and RCA-I from *Ricinus communis*) and Moraceae (jacalin and the jacalin-related lectin MPA from *Maclura pomifera*). A few T/Tn-specific lectins such as SNA-I and SNA-V from the elderberry *Sambucus nigra*, BGSL from the bitter gourd (*Momordica charantia*), and the galactose-specific lectin and ricin from the castor bean (*Ricinus communis*) correspond to chimerolectins composed of a toxic A-chain covalently linked to a B-chain displaying the T/Tn-specificity [28]. Fungal lectins (ABL from *Agaricus bisporus*, AAL from *Agrocybe aegerita* and XCL from *Xerocomus chrysenteron*) with a T/Tn-specificity belong exclusively to the group of Basidiomycota mushrooms. Although most of the so-called T/Tn-specific lectins readily react with both T and Tn antigens, T and Tn consist of very distinct antigens that arise by different mechanisms and in different cancerous tissues. In addition, plant lectins specific for T and/or Tn antigens also interact with α-d-Gal and the Gal-specificity of some T/Tn-specific lectins such as jacalin from *Artocarpus integrifolia* seeds is as potent and even stronger than that displayed for both T and Tn antigens [29,30]. This stronger interaction with α-d-Gal depends on its fixation to the primary binding site of the lectin via a dense network of nine hydrogen bonds, as shown from the crystallographic complex of jacalin with galactose (PDB code 1UGW) [31].

The carbohydrate-binding site (CBS) of plant and fungal lectins consists of a carbohydrate-binding pocket, the so-called monosaccharide-binding site, responsible for the binding of simple sugars via a network of hydrogen bonds linking the sugar to a few polar residues forming the binding pocket [23]. Usually, additional non-polar stacking interactions with aromatic residues located in the close vicinity of the monosaccharide-binding site, complete the anchorage of simple sugars to the site. The area surrounding the monosaccharide-binding site delineates an extended carbohydrate-binding site, which also participates in the binding of more complex glycans by creating additional H-bonds and stacking interactions with other sugar units of the glycan chain. In fact, the CBS consists of a monosaccharide-binding site embedded in a more extended binding area forming the extended glycan-binding site. Due to the extreme variation in the shape and size of the extended CBS from one lectin to another, the tight association of the monosaccharide-binding site and extended binding site, offers to plant and fungal lectins an extremely versatile tool for the specific recognition of complex *O*-glycan chains. Such a versatility explains why different lectins displaying the same broad sugar specificity, i.e., the recognition of a unique simple sugar such as Gal, GalNAc or Man, often differ by their ability to specifically recognize more complex glycans, depending on different shape and size of their extended CBS [23].

**Figure 3:**
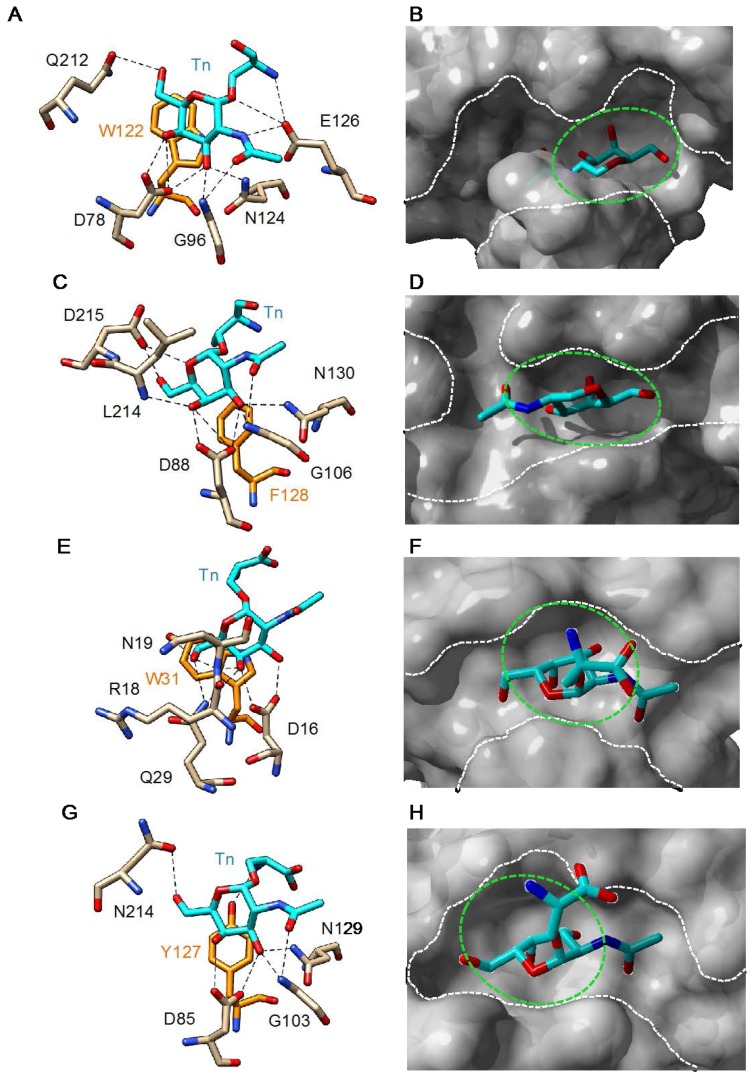
(**A**,**C**,**E**,**G**) Network of hydrogen bonds and stacking interactions anchoring Tn antigen (Tn) to the monosaccharide-binding site of: *Bauhinia forficata* BfL lectin (**A**) (PDB code 5T5J) [46]; soybean lectin SBA (**C**) (PDB code 4D69) [89]; *Sambucus nigra* SNA-II lectin (**E**) (PDB code 3CA6) [74]; and *Vicia villosa* VVA-B_4_ lectin (**G**) (PDB code 1N47) [90]. Amino acid residues involved in stacking interactions with the disaccharide are colored orange; (**B**,**D**,**F**,**H**) Docking of Tn antigen to the monosaccharide-binding cavity (green dashed circle) of: *Bauhinia forficata* BfL lectin (**B**); soybean lectin SBA (**D**); *Sambucus nigra* SNA-II lectin (**F**); and *Vicia villosa* VVA-B4 lectin (**H**). The white dashed lines delineate the extended binding sites at the molecular surface of the different lectins. Cartoons drawn with Chimera [91].

All of the T/Tn-specific lectins consist of dimeric or tetrameric structures built up from the non-covalent association of identical monomers, except for the B-chain lectins of type 2 RIPs (abrin and ricin) which consist of a single polypeptide chain arranged in two domains. Plant and fungal Tn-specific lectins accommodate the Tn antigen in the monosaccharide-binding site, which consists of a shallow pocket located at the top of each lectin monomer or domain. The binding of Tn antigen is achieved by a network of hydrogen bonds between the oxhydryls of the GalNAc residue and a few hydrophilic amino acids. Stacking interactions between the GalNAc ring and aromatic residues located in the vicinity of the monosaccharide-binding site, complete and reinforce the binding of Tn antigen to the lectins (Figure 3). Depending on the lectins, hydrogen bonds can occur between the amino acid moiety of the Tn antigen and the CBS of the lectin (Figure 3A). Although the amino acid residues forming the monosaccharide-binding site of Tn-specific lectins markedly differ from one lectin to another, the binding scheme of Tn antigen to the lectins remains very similar with O3, O4, O6 and O7 of GalNAc systematically involved in H-bonds with the amino acids of the site. However, some discrepancy can occur with the number of hydrogen bonds that link the Tn antigen to the monosaccharide-binding pocket, which varies from 7 to 10, depending on the lectin (Figure 3A,C,E,G). Accordingly, the affinity toward the Tn antigen should slightly differ among the different Tn-specific lectins of plant and fungal origin. In this respect, an interesting observation was reported by Osinaga et al. [86], who showed that VVL-B_4_, the Tn-specific *Vicia villosa* lectin, binds a single Tn epitope in surface plasmon resonance spectroscopy experiments, whereas the anti-Tn moAbs 83D4 and MLS128 only recognize two consecutive Tn epitopes. These results point out the importance of Tn clusters for the correct binding of moAbs and suggest a very different Tn recognition pattern for VVLB4 and the anti-Tn moAbs. Moreover, the recent observation by [87], that some selectivity in the binding of lectins to Tn antigen depends on the nature of the amino acid residue (Ser or Thr) linked to α-*O*-GalNAc, brings an additional complexity to the glycan-binding mechanisms of plant lectins.

**Figure 4:**
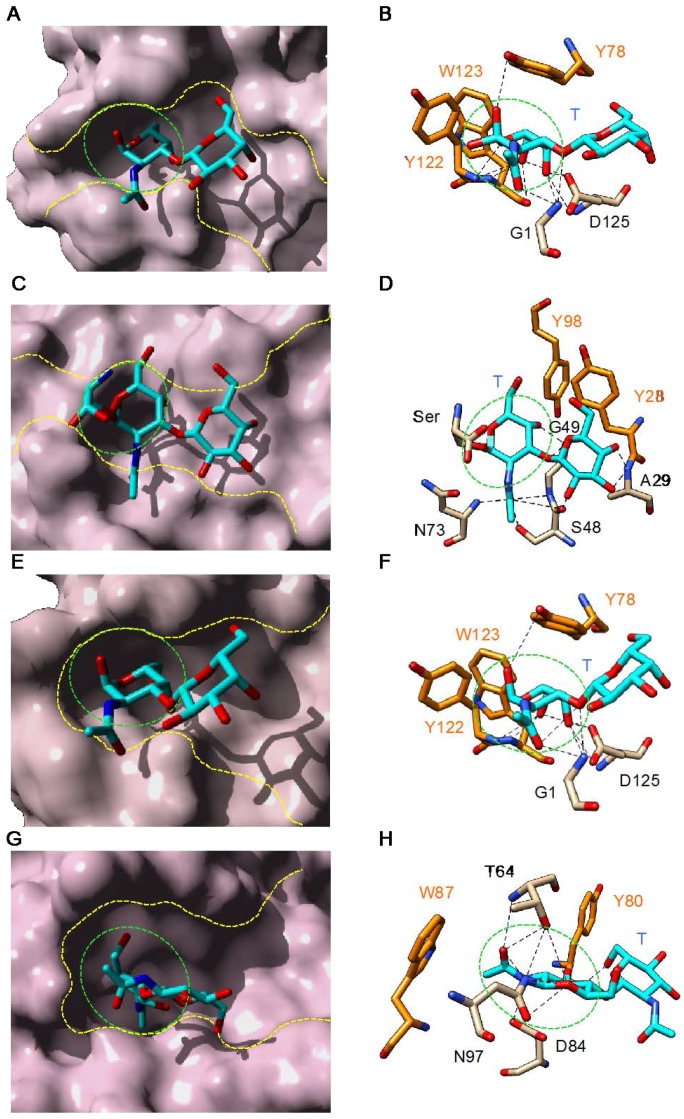
Monosaccharide-binding sites (green dashed circles) and extended binding sites (yellow dashed lines) of: jacalin (*Artocarpus integrifolia*) (PDB code 1M26) [92] (**A**); the mushroom *Agaricus bisporus* lectin ABL (PDB code 1Y2V) [34] (**C**); the Osage orange (*Maclura pomifera*) lectin MPA (PDB code 1JOT) [58] (**E**); and the bitter gourd (*Momordica charantia*) galactose-specific lectin BGSL (PDB code 4ZGR) [62] (**G**), in complex with T-antigen (Galβ1→3GalNAcα1→Ser/Thr). Network of hydrogen bonds (dashed lines) anchoring T-antigen (colored cyan) to the amino acid residues of the extended binding site of: jacalin (**B**); ABL (**D**); MPA (**F**); and BGSL (**H**). Amino acid residues involved in non-polar stacking interactions with the disaccharide are colored orange. Cartoons drawn with Chimera [91].

The binding of the Thomsen–Friedenreich T-antigen (Galβ1→3GalNAcα1→Ser/Thr) to the plant and fungal T-specific lectins looks very similar to the binding of Tn antigen (Figure 4). The disaccharide becomes anchored to the CBS of the lectins through a network of hydrogen bonds associated with non-polar stacking interactions with aromatic residues. Most of the H-bonds occur with the GalNAc residue, which occupies the carbohydrate-binding pocket of the lectins. Usually, the ultimate Gal residue of the disaccharide remains unbonded and protrudes out of the extended CBS. However, in some lectins such as the mushroom *Agaricus bisporus* lectin ABL (Figure 4D) and the bitter gourd (*Momordica charantia*) galactose-specific lectin BGSL (Figure 4H), a very limited number of H-bonds can occur between the Gal residue and amino acid residues of the extended CBS. With the exception of these few lectins, the role played by the extended CBS in the binding of the disaccharidic *O*-glycans is apparently negligible.

Obviously, the binding of T and Tn antigen is restricted to the carbohydrate-binding pocket of the plant and fungal T/Tn-specific lectins. The surface area located in the neighbor of the CBS, which forms the extended carbohydrate-binding site of the lectins, does not participate in the binding of both antigens. However, the extended CBS participates in the binding of more extended *O*-saccharides, e.g., trisaccharides such as the Lewis a (Galβ1→3[Fucα1→4]GlcNAcβ1→R), and Lewis x (Galβ1→4[Fucα1→3]GlcNAcβ1→R) antigen, as shown from the crystallographic complex of GS4 lectin of *Griffonia simplicifolia* with the methyl-glycoside of the Lewis b blood group determinant (Figure 6) [88]. Up to 18 hydrogen bonds anchor the three sugar residues of Lewis b to the extended CBS of the lectin, in association with extensive non-polar stacking interactions with five aromatic residues (Y105, F108, W133, W138, and Y223). The ultimate Gal residue of the trisaccharide binds to the carbohydrate-binding pocket via four H-bonds, whereas the remaining 14 H-bonds serve to anchor the Fuc and GlcNAc residues of the trisaccharide to amino acids located in the extended CBS. The shape and size of the extended CBS thus appears as a determinant structural feature for the recognition of extended *O*-glycans by plant lectins. In this respect, plant lectins readily differ from monoclonal antibodies used as probes for targeting the complex *O*-glycans (≥3 sugar residues) of cancer cells, which usually recognize a limited portion of the *O*-glycan chain.

**Figure 5:**
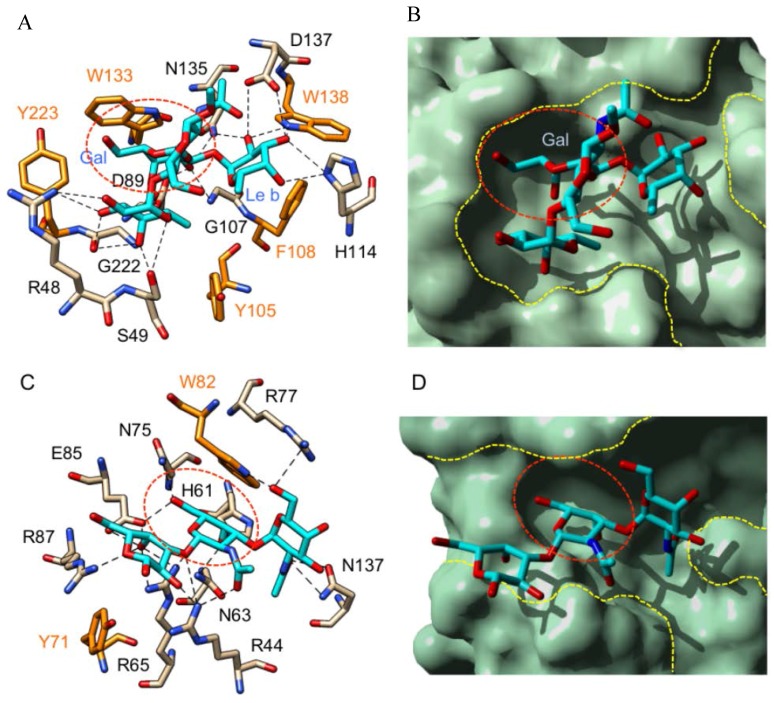
(**A**) Network of hydrogen bonds (dashed lines) anchoring Lewis b tetrasaccharide (colored cyan) to the amino acid residues of the monosaccharide-binding site (red dashed circle) of Gs I-A_4_ (*Griffonia simplicifolia*) (PDB code 1LED) [88]. Amino acid residues involved in stacking interactions with the trisaccharide are colored orange. The Gal residue (Gal) of the Lewis b antigen occupies the monosaccharide-binding pocket of the lectin; (**B**) Molecular surface of Gs I-A_4_ showing the monosaccharide-binding site (red dashed circle) and the extended binding site (yellow dashed lines) complexed to the Lewis b trisaccharide. The Gal residue (Gal) of the Lewis b antigen occupies the monosaccharide-binding pocket of the lectin; (**C**) Network of hydrogen bonds (dashed lines) anchoring the Forssman trisaccharide (colored cyan) to the amino acid residues of the carbohydrate-recognition domain of galectin-9 (PDB code 2EAL) [93]. Amino acid residues involved in stacking interactions with the trisaccharide are colored orange. The red dashed circle delineates the monosaccharide-binding site of the lectin; and (**D**) Molecular surface of galectin-9 showing the monosaccharide-binding pocket (red dashed circle) and the extended binding site (yellow dashed lines) complexed to the Forssman trisaccharide. The penultimate GalNAc residue (GalNAc) of the Forssman antigen occupies the monosaccharide-binding pocket of the lectin. Cartoons drawn with Chimera [91].

A similar binding pattern was shown to occur in galectin-9 complexed to the Forssman antigen (PDB code 2EAL) [93]. A network of 17 H-bonds anchors the trisaccharide moiety of the Forssman antigen to the carbohydrate-recognition domain of galectin-9 and seven H-bonds serve to anchor the penultimate GalNAc residue of the Forssman antigen, which occupies the monosaccharide-binding site of the galectin (Figure 5C,D). Two non-polar stacking interactions with two aromatic residues (Y71,W82) of the extended CBS complete the binding of the Forssman antigen to the lectin. According to their capacity to accommodate extended *O*-glycan chains, both galectins and plant lectins similarly differ from monoclonal antibodies used as probes, which usually recognize a more limited portion of the *O*-glycan chain.

## 4. Tn/T-Specific Lectins for Cancer Diagnosis/Prognosis

Lectin histochemistry using T/Tn-specific lectins was previously investigated for the screening of the glycosylation changes occurring at the surface of cancer cells. Targeting of T and Tn markers by lectins proved to be an efficient tool for both the detection and prognosis of many cancers [94,95,96]. Especially, lectin binding pattern of peanut (PNA) and horse gram (DBA from *Dolichos biflorus*) agglutinins, were used as histochemical probes to determine the malignant status of both oral and colonic mucosa [97,98,99,100,101,102]. Moreover, the density of Tn antigen at the cell surface appeared as a good predictor of the aggressiveness in primary breast carcinoma [103]. The increase of T antigen occurrence in cancer cells often correlates with cancer progression and metastasis development [104]. Peanut lectin PNA strongly reacted with follicular carcinoma cells, whereas soybean agglutinin SBA, *Griffonia simplicifolia* lectin GSL and *Vicia villosa* agglutinin VVA reacted with cells lining the papillary structures in papillary carcinomas of the thyroid gland [105]. Around one third of breast cancer tumors displayed a strong binding of the mistletoe (*Viscum album*) lectin ML-I (VVA-1) and statistics indicated an inverse correlation between disease outcome and lectin binding [106]. Changes in *O*-glycosylation at different stages of differentiation of cervical intraepithelial dysplasia were investigated using different lectins [107]. The *Amaranthus caudatus* T/Tn-specific amaranthin, showed an increased reactivity towards dysplasia cells at stage II whereas SBA (Tn-specific soybean lectin) and GS4 (Tn-specific *Griffonia simplicifolia* lectin), did not discriminate among the different stages of dysplasia cells and normal tissue cells. The epithelial mesenchymal transition of HGF-treated Huh7 hepatocellular carcinoma cells is associated with a decreased affinity for a panel of T/Tn-specific lectins including ACL (*Amaranthus caudatus*), BPL (*Bauhinia purpurea*), jacalin, SBA (soybean) and SNA (*Sambucus nigra*) [108]. These results imply that glycan structures containing T and Tn antigens exposed at the cell surface of hepatocellular carcinoma cells are drastically reduced during the epithelial mesenchymal transitions of the cells, suggesting a pivotal role for the cell surface *O*-glycan transformations in tumor metastasis.

**Figure 6:**
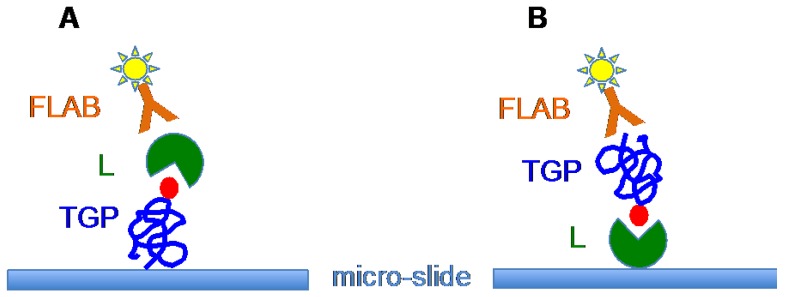
(**A**) Glycoprotein-microarray technology showing the spotted tumor glycoprotein (TGP) recognized by the lectin probe (L) and visualized by a fluorescent-labeled anti-lectin antibody (FLAB). (**B**) Lectin-microarray technology showing the spotted lectin (L) recognized by the tumor glycoprotein probe (TGP) and visualized by a fluorescent-labeled anti-glycoprotein antibody (FLAB) (adapted from [114]).

The use of plant and fungal lectins as cancer biomarkers has been greatly improved by the recent introduction of the glycoprotein-microarray and lectin-microarray technologies [109,110,111,112,113,114,115,116]. Glycoprotein-microarray technology consists of glycan structures isolated from the tumor cell surface, arrayed on micro-slides, and subsequently probed with individual lectins (Figure 6A). In the lectin-microarray technology, a set of different lectins is spotted on micro-slides and subsequently probed with membrane glycoproteins isolated from the tumors (Figure 6B). In both technologies, a fluorescently labeled antibody allows the recognition of the molecules used as probes. Both approaches allow screening of a number of tumor samples and may be used for the early diagnosis of cancer, and the follow up of tumor progression and their recurrence [115].

These technologies also apply to the detection of serum cancer biomarkers and have been used widely for the serum glycoprotein profiling for colorectal cancer [117,118], pancreatic cancer [119], and also for breast cancer using the related approach of multilectin affinity chromatography coupled to HPLC-tandem mass spectrometry [120]. A lectin/glyco-antibody microarray performed on sera from 22 pancreatic cancer sera, discriminated cancer patients from other diseases (35 chronic pancreatitis, 37 diabetes) and normal controls (89 controls), by a 70% increase inthe response of serum α1β-glycoprotein to SNA (*Sambucus nigra* agglutinin) [121]. Using a 45-immobilized lectin microarray, the cell surface glycan changes occurring during the malignant transformation of endometrium, were investigated [122]. Depending on the glycan profile for six lectins including the T/Tn-specific lectins SNA (*Sambucus nigra*), ACA (*Amaranthus caudatus*) and BPL (*Bauhinia purpurea*), the different stages identified in the malignant transformation could be distinguished. Interestingly, cell lines exhibiting the higher anticancer drug-resistance displayed the stronger binding to three lectins (ACA, BPL and the *Dolichos biflorus* lectin DBL), whereas drug-sensitive cell lines had almost no activity for the lectins. Accordingly, glycan profiling with an adapted lectin-microarray should allow the lectin-microarray technology to predict the success of chemotherapy with selected anticancer drugs. Using a panel of 37 lectins immobilized on a lectin microarray, the normalized fluorescence intensity measured with T/Tn-specific lectins (jacalin, MPL from *Maclura pomifera*, DBA from *Dolichos biflorus*, ACA from *Amaranthus caudatus* and VVA from *Vicia villosa*) measured for Cy3-labeled gastric cancer cell glycoproteins was stronger compared to the fluorescent intensity measured for Cy3-labeled gastric ulcer cell glycoproteins [123]. Similar results were obtained upon staining of paraffin-embedded gastric cancer and gastric ulcer tissues with MPL and VVA. Similarly changes in the glycan profile of sialylated MUC1 in cholangiocarcinoma were investigated using a 43-lectin-immobilized microarray [124]. Interaction with immobilized WFA (*Wisteria floribunda* agglutinin) alone proved to be sufficient to discriminate between cholangiocarcinoma and hepatholithiasis. A lectin-based microarray analysis discriminated between healthy patients and patients with colorectal cancer due to the higher degree a α2,6-sialylation and the higher content in high mannose *N*-glycans in serum α2-macroglobulin [125]. Glycosylation profiling of fibronectin performed with a lectin-microarray, showed that PNA distinguishes between two distinct types of non-small cell lung carcinomas, lung adenocarcinoma and large cell lung carcinoma [126]. In a lectin-microarray analysis including the T/Tn-specific lectins ACA (*Amaranthus caudatus*), ACG (*Agrocybe cylindracea*), BPL (*Bauhinia purpurea*), and SNA (*Sambucus nigra*), a decreased lectin-binding activity was observed for Tn antigen from formalin-fixed human choriocarcinoma tissues [24]. Using a panel of 17 lectins including T/Tn-specific lectins (ACA, PNA, SNA, VVL, and WFA) immobilized on a microarray integrated on a microfluidic lab-on-a-chip platform, distinct signature glycoprofiles were established for sera and tissue samples from gastritis and gastric cancer patients [127]. A lectin microarray approach using a number of immobilized T/Tn-specific lectins including jacalin, ABL (*Agaricus bisporus*), BPL (*Bauhinia purpurea*), GSA (*Griffonia simplicifolia*), MPA (*Maclura pomifera*), SBA (soybean), RCA120 (castor bean), VVA (*Vicia villosa*) and WFL (*Wisteria floribunda*), was successfully applied to formaline-fixed tumor samples to identify the metastasis-associated changes in glycosylation profiling of breast cancer cells [128,129]. Using a lectin microarray and LC-MS/MS approach, PNA (*Arachis hypogaea*) interacted with HSR-GBM1 and U373 glioblastoma cell lines and was used to capture and characterize the corresponding PNA-binding glycoproteins [130].

The prognostic value of the lectin histochemistry was investigated in various cancer diseases. Expression of the Tn antigen in different types of breast cancer detected by VVA-B_4_ lectin, developed very early, before any differentiation and destructive changes become detectable [131]. The binding of VVA-B_4_ to primary cancer cells was attributed to the Tn-antigen-bearing MUC1 protein in primary breast cancer in relation to lymphatic metastasis [132]. Binding of PNA (peanut agglutinin) to a CD44 variant glycoprotein receptor in HT29 colon cancer cells, correlated with an increased metastatic potential [133]. A positive PNA binding to lung adenocarcinoma cells in both the primary tumor and the nodal lesions, was associated with a significantly higher survival rate of patients [134]. Low binding of BPL, the T/Tn-specific *Bauhinia purpurea* lectin, was identified as a predictive factor for the recurrence of gastric cancer in gastrectomized patients (*n* = 60), in association to lymph node metastasis [135]. An attenuated VVA (*Vicia villosa* agglutinin) binding to metastatic lymph node cells was also observed in advanced gastric cancer as compared to the strong recognition of the lectin by primary advanced gastric cancer cells [136]. In addition, the histological type of advanced gastric cancer was strongly associated with the binding of soybean lectin (SBA) and *Bauhinia purpurea* lectin (BPL), except for the p53 mutations which correlate well with the *Griffonia simplicifolia* lectin II (GSA). Conversely, a lower histochemical binding of AAL, the sialyl T-specific lectin from the mushroom *Agrocybe aegerita*, has been identified as a significant favorable prognostic factor for the free survival in colorectal cancer [137]. In triple-negative breast cancer, tissue microarray showed that binding of RCA-I, the castor bean (*Ricinus communis*) lectin, to cancer cells correlated with the TNM grades, suggesting that RCA-I-specific glycoproteins of cell surface play a critical role in metastasis [138]. A strong RCA-I binding was associated with a strong incidence of developing metastases in triple-negative breast cancer patients. A lower binding of ACA, the *Amaranthus caudatus* lectin, to gastric cancer cells correlated with poorer patient prognosis in integrated lectin-microarray and mass sprectrometry analyses [139]. Higher values of serum *Wisteria floribunda* agglutinin-positive Mac-2 binding protein (WFA+-M2BP) were associated with the risk for development of hepatocellular carcinoma among patients with chronic hepatitis C after sustained virological response by interferon treatment [140].

## 5. Toxic Effects of Tn/T-Specific Lectins on Cancer Cells

The non-exhaustive Table 2 shows the extreme diversity of cancer cell lines that have been addressed to probe the in vitro toxicity of plant and fungal T/Tn-specific lectins towards transformed cells.

Depending on the lectins, the toxic effect of T/Tn-lectins on cancer cells results in cell death, primarily via apoptosis induction or more or less severe inhibition of the proliferative capacity of cancer cells, or both [17,35,141,142,143,144,145]. Autophagy was also incriminated as a toxic effect of T/Tn-lectins on cancer cells [146]. In addition, the mechanism(s) underlying the cytotoxicity of T/Tn-specific lectins belonging to the type 2 Ribosome-Inactivation Proteins (RIPs), e.g., ricin, abrin and the mistletoe (*Viscum album*) lectins, partly differ from that of hololectins by the occurrence of a toxic A-chain acting as a potent protein biosynthesis inhibitor, which inhibits the ribosomal protein biosynthesis by depurinating the adenine base at position 2543 of the rRNA 28S [147]. The inhibition of protein synthesis due to the abrin-A chain, thus appears as the major determinant for the cytotoxicity of type 2 RIPs [148], the abrin-B lectin chain allowing the recognition of abrin by the target cell via a specific interaction with membrane *O*-glycoproteins resulting in the subsequent internalization of the toxic A-chain into the cell. However, recent results comparing the toxic effects of different elderberry (*Sambucus nigra*) type 2 RIPs (SNA-I, SNA-V, and SNRLP) and non-RIP lectins (SNA-II and SNA-IV), point toward a toxic effect of the B-chain lectin that most probably involves an autophagy induction-pathway, in addition to the toxic effect of A-chain on protein biosynthesis [146].

Following their recognition by the cancer cell membrane receptors, T/Tn-lectins can regulate a number of signaling pathways responsible for the apoptotic, anti-proliferative and autophagic effects on the cancer cells in vitro and in vivo. Table 3 summarizes some of these pathways used by T/Tn-lectins to induce apoptotic and autophagic effects, and inhibition of the proliferation on cancer cells.

Recent investigations using proteomic approaches including network construction, hub protein identification, targeted microRNA prediction and microarray analyses, pointed out the occurrence of the extreme diversity of signaling pathways associated to apoptosis and DNA modifications in the lectin-treated cancer cells. The Chinese mistletoe lectin-I CMI induced apoptosis in colorectal cancer cells CLY and HT-29 by down-regulating miR-135a&b expression and up-regulating expression of their APC (Adenomatous Polyposis Coli) target gene [149]. Nine autophagic hub proteins and 13 tumor suppressive miRNA were identified in plant lectin-treated breast carcinoma MCF-7 cells [150]. Using mRNA- and miRNA-microarrays, the SRL (T/Tn-specific *Sclerotum rolfsii* lectin) treatment of HT29 colon cancer cells resulted in the altered expression of several hundred proteins including MAPK, c-JUN, apoptosis-associated and DNA replication-associated signaling molecules [151]. More recently, up to 22 apoptotic hub proteins were identified in the global human protein-protein interaction network built up for lectin-treated mesothelioma cells according to their different microarray expression, together with the miRNA predicted to negatively regulate these hub proteins [152].

Despite the cytotoxic effects on both healthy and malignant cells, some T/Tn-specific lectins display a pronounced mitogenic effect susceptible to improve the proliferation of cancer cells. At low concentrations, peanut lectin stimulates the proliferation of colon cancer cell lines (HT29, T84, Caco2) due to the activation of the hepatocyte growth factor c-Met and the resulting activation of MAPK [27]. The tumor growth of BALB/c mice fed with daily doses of *Agaricus bisporus* lectin ABL, was significantly enhanced as compared with control mice, suggesting an immunomodulatory effect of the lectin that reduces the innate and adaptive responses of the cells [186]. Upon fixation at the cell surface, T/Tn-specific lectins can activate signaling pathways responsible for the production of cytokines susceptible to induce an immune response against tumor cells. Mistletoe (*Viscum album*) lectin ML-I induces the production by human PBMCs (Peripheral Blood Mononuclear Cells) of a set of IL-6, IL-10, IL-12 and TNFα [187,188,189]. The Korean mistletoe lectin also induces the secretion of IL-12 by human dendritic cells [190]. However, the immunomodulatory effects of plant and fungal lectins do not rely on the T/Tn-binding specificity since many other lectins with different carbohydrate-binding specificies, for example legume lectins such as PHA-L (*Phaseolus vulgaris*) [191] or ArtiM from *Artocarpus heterophyllus* [192], also display immunomodulatory properties.

## 6. Tn/T-Specific Lectins as Targeting Aids for the Photodynamic Treatment of Tumors

**Figure 7:**
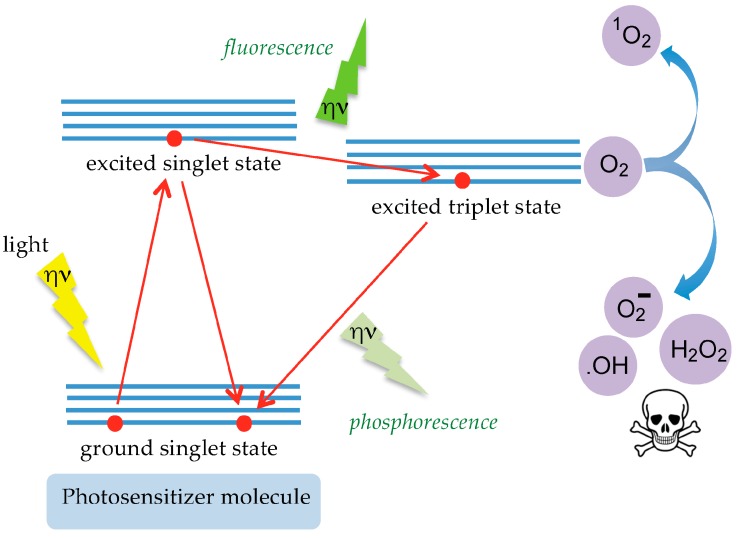
Mechanism of action of photosensitizers. Upon illumination at a selective wavelength (light), the photosensitizer becomes excited (excited singlet state) and reaches, after relaxation, a steady-excited state (excited triplet state) for a longer duration associated with the emission of fluorescence. Collisions with O_2_ produce different forms of active oxygen (O_2_^−^, **^·^**OH, and H_2_O_2_) able to kill the cancer cells.

The local treatment of tumors using photodynamic therapy (PDT), uses photosensitizers which, upon illumination at a specific wavelength, become activated and produce different forms of active singlet oxygen known as ROS (Reactive Oxygen Species) that kill the tumor cells (Figure 7). Following its injection in the bloodstream or directly into the tumor, the photosensitizer is equally absorbed by healthy and tumor cells but it disappears faster from healthy cells, compared to tumor cells. Accordingly, the laser lightening of the tumor is performed 48 to 72 h after the injection of the photosensitizer, to ensure that cancer cells will be killed without harming the neighboring healthy cells.

**Figure 8:**
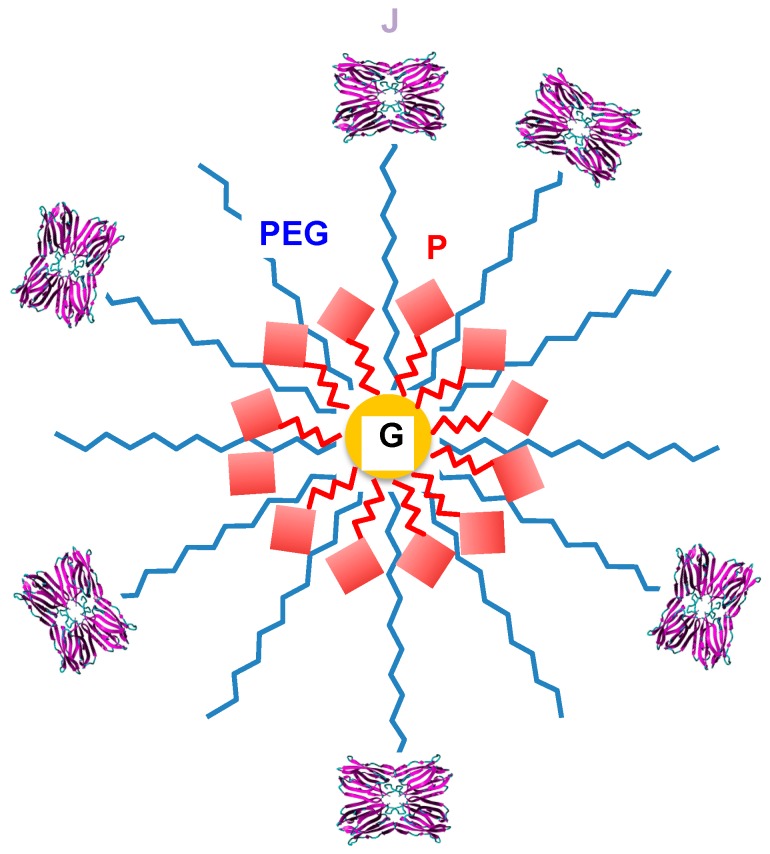
Lectin conjugated phthalocyanine-PEG gold nanoparticle made of a gold nanoparticle (G) covered with Zn phthalocyanine molecules (P) and polyethylene glycol (PEG) covalently linked to jacalin molecules (J) (adapted from [207]).

Although most of the photosensitizing molecules used for PDT stay longer in cancer cells compared to healthy cells, their combination with other molecules that specifically recognize the receptors located at the cell surface, greatly enhances the targeting of photosensitizers to the tumors. Due to the widespread distribution of aberrant *O*-glycans on the surface of most cancer cells, T/Tn-specific lectins have been identified early as potential targeting molecules available for PDT [193,194]. The non-covalent binding between porphyrins and different T/Tn-specific lectins including jacalin from the Jackfruit (*Artocarpus integrifolia*) [195], the snake gourd (*Trichosanthes aguina*) [196], and the bitter gourd (*Momordica charantia*) [197], was investigated. The binding of lectins to porphyrin and phycobilin molecules was further extended to the T/Tn-specific lectin PNA [198] and to other legume lectins such as the mannose-specific concanavalin A [199] and the garden pea lectin [200]. The interaction of jacalin with phycobilin, another photosensitizing molecule, was also studied [201]. However, all of these complexes resulting from the non covalent interaction of lectins with porphyrins and phycobilins, exhibited a rather weak stability in in vitro experiments performed with cancer cell lines, suggesting that T/Tn-specific lectins require to be covalently bound to photosensitizing molecules to properly target the cancer cells [202]. In a series of experiments, Poiroux et al. [203,204,205], showed the relevance of the covalent binding of Morniga G, the T/Tn-specific lectin from the mulberry (*Morus nigra*), to porphyrins and phthalocyanines to improve both the stability of the lectin-photosensitizer complexes and their efficacy to kill Jurkat leukemia cells. A more sophisticated conjugation of targeting molecules (antibodies and lectins) to photosensitizers using the drug carriers Zn-porphyrin-cyclodextrins was subsequently introduced by Kejík et al. [206] as a versatile delivery system for anticancer drugs. Recently, another approach based on the conjugation of jacalin to phthalocyanine-PEG gold nanoparticles (4 nm in diameter), was developed by Obaid et al. [207,208], to selectively target and destroy the HT-29 human colon and the SK-BR-3 human breast adenocarcinoma cells. The conjugates consist of gold nanoparticles covered by a monolayer of Zn phthalocyanine and polyethylene glycol (PEG) further functionalized with jacalin (ca. 6 jacalin molecules bound to each gold nanoparticle) (Figure 8).

The advantage is that this conjugation technique enables a higher concentration of photosensitizers at the surface of the tumor cells, susceptible to improve the efficiency of PDT. Once anchored to the cell surface by the lectin moiety, the conjugates are further endocytosed into the cells and produce singlet oxygen forms upon illumination at the wavelength corresponding to the activation of Zn phthalocyanin. The possible use of lectin-coated nanoparticles to specifically target tumors and their microvascular environment has recently been argued as new anticancer therapeutic opportunities [209]. Obviously, the ability of plant and fungal lectins to be endocytosed quickly following the specific recognition of glycans at the cancer cell surface, make them good candidates to build photosensitizer conjugates adapted to PDT. In this respect, FITC-labeled Morniga G, the T/Tn-specific lectin from black elderberry *Morus nigra*, was shown to readily enter Jurkat cells within 5 min of incubation at 37 °C, upon fixation at the cell surface [210]. Recently, another approach based on the conjugation of galactodendrimers to phthalocyanine was proposed by Pereira et al. [211], to specifically target the carbohydrate-binding receptors occurring on the tumor cells, instead of the aberrant *O*-glycans exposed at the tumor cell surface.

To date, PDT is restricted to the treatment of superficial skin tumors and tumors located in the body cavities such as esophageal cancer or non-small cell lung cancer [212]. However, the progress achieved in the photosensitizer efficacy and specificity towards cancer cells should expand the use of PDT to other cancers, e.g., cancers of the peritoneal cavity, brain and prostate. The improvement of the laser source equipment that delivers the activating light to the photosensitizing molecules absorbed by the tumor is also of paramount importance in order to apply PDT to the treatment of deeper and larger tumors [213,214]. In this respect, the use of new photosensitizer molecules activatable upon illumination at red and infrared wavelengths greatly enhances the efficacy of the PDT due to a deeper penetration of more efficient red and infrared wavelengths into the malignant cells.

## 7. Conclusions

Plant and fungal lectins displaying a T/Tn-specificity have been widely used as relevant probes for the histochemical detection of aberrant *O*-glycan glycomarkers expressed at the surface of malignant cells. With the aid of the fast-developing glycan- and lectin-microarray technologies, our increasing knowledge on the fine carbohydrate-binding specificity of plant and fungal lectins has revealed the extreme versatility of the lectin tool to specifically recognize discrete/subtle differences in the expression of altered glycans by cancer cells. Depending on the lectins, the affinity towards complex *O*-glycans and their chemical substitutions such as sialylation or sulfation can vary in a large proportion. The ability of T/Tn-specific lectins to accommodate large *O*-glycans to the extended carbohydrate-binding site via a complex network of hydrogen bonds and hydrophobic interactions, accounts for such versatility. Variations in the shape and size of the extended carbohydrate-binding site from one lectin to another, readily explain the discrepancies observed in the binding activity among different T/Tn-specific lectins. Compared to monoclonal antibodies used as standard probes for the detection of the *O*-glycosylation aberrations occurring at the cancer cell surface, plant and fungal T/Tn-specific lectins consist of a complementary sugar-recognition domain that is equally performant, if not more, in the recognition of complex *O*-glycans. Their flexibility in the recognition of complex *O*-glycans, make T/Tn-specific lectins good candidates available for the specific targeting of aberrant *O*-glycans in the photodynamic treatment of cancer. In vitro experiments performed with Morniga-G, the T/Tn-specific lectin of elderberry (*Morus nigra*), demonstrated the feasibility to covalently attach a plant lectin to photosensitizers, the subsequent specific recognition and engulfment by transformed cells, and the selective killing of the sole transformed cells [203,204,205]. These stimulating results pave the way for the use of carefully selected T/Tn-specific lectins as targeting molecules for the photodynamic treatment of cancers.

## Figures and Tables

**Table 1:** List of the plant (P) and fungal (F) Tn/T-specific lectins.

Plant/Fungus	Lectin	Specificity	References
*Abrus precatorius* (P)	APA	T	[32]
*Agaricus bisporus* (F)	ABL	T	[33,34]
*Agrocybe aegerita* (F)	AAL	ST/T	[35]
*Agropyrum repens* (P)	ARL	T	[36]
*Amaranthus caudatus* (P)	Amaranthin	T/Tn	[37,38]
*Amaranthus leucocarpus* (P)	ALL	T/Tn	[39]
*Arachis hypogaea* (P)	PNA	ST > T > Tn	[38,40,41]
*Artocarpus incisa* (P)	Frutalin	T	[42]
*Artocarpus integrifolia* (P)	Jacalin	ST/T/Tn	[43]
	Champedak GBL	Tn of O-mucin	[44]
*Artocarpus lakoocha* (P)	ALL	T/Tn cluster	[45]
*Bauhinia forficata* (P)	BfL	Tn	[46]
*Bauhinia purpurea* (P)	BPA	T/Tn cluster	[47]
*Caragana arborescens* (P)	CAA	ST, Forssman	[48]
*Codium fragile* (alga)	CFA	T/Tn, Forssman	[49]
*Dolichos biflorus* (P)	Tn	Tn	[50]
*Glechoma hederacea* (P)	Gleheda	T/Tn	[51]
*Glycine max* (P)	SBA	Tn, mucin	[52]
*Griffonia* (*Bandeirea*) *simplicifolia* (P)	Gs I-A_4_	Tn	[53,54]
*Lactarius deliciosus* (F)	LDL	T	[55]
*Lactarius deterrimus* (F)	LDetL	T	[56]
*Laelia autumnalis* (P)	LAL	T/Tn	[57]
*Maclura pomifera* (P)	MPA	T/Tn	[58,59]
*Moluccella laevis* (P)	MLL	Tn, Forssman	[60,61]
*Momordica charantia* (P)	BGSL	T	[62]
*Morus nigra* (P)	Morniga-G	Tn/T cluster	[63]
*Myrsine coriacea* (P)	McL	Tn	[64]
*Psophocarpus tetragonolobus* (P)	WBL	Tn	[65]
*Ricinus communis* (P)	Ricin	T/Tn	[66]
	RCA-I	T	[67]
*Salvia bogotensis* (P)	SBL	Tn	[68]
*Salvia sclarea* (P)	SSL	Tn	[69,70,71]
*Salvia hominum* (P)	SHL	Tn	[72]
*Sambucus nigra* (P)	SNA	Tn cluster	[73]
	SNA-II	Tn	[74]
	SNA-IV	Tn	Unpublished
*Sclerotium rolfsii* (F)	SRL	Tn cluster	[75]
*Sophora japonica* (P)	SJL	T	[76]
*Triticum vulgare* (P)	WGA	Tn cluster	[77,78]
*Vateirea macrocarpa* (P)	VML	T/Tn	[79]
*Vicia graminea* (P)	VguL	T	[80]
*Vicia villosa* (P)	VVA B_4_	Tn	[81]
*Viscum album* (P)	ML-I	T	[82]
*Wisteria floribunda* (P)	WFA	Tn	[83]
*Xerocomus chrysenteron* (F)	XCL	Tn	[84]
*Ximenia americana* (P)	Riproximin	Tn cluster	[85]

**Table 2:** In vitro cytotoxicity and inhibition of proliferation of cancer cell lines by T/Tn-specific lectins (ABL: *Agaricus bisporus* lectin; AAL: *Agrocybe aegerita* lectin; BfL: *Bauhinia forficata* lectin; GSA-IA4: *Griffonia simplicifolia* lectin; jacalin (*Artocarpus integrifolia* lectin); McL: *Myrsine coriacea* lectin; MCL: *Momordica charantia* lectin; ML-I, ML-II, ML-III: Mistletoe (*Viscum album*) lectins; PNA: peanut (*Arachis hypogaea*) agglutinin; ricin: *Ricinus communis* lectin; SBA: soybean (*Glycine max*) agglutinin; SRL: *Sclerotium rolfsii* lectin; riproximin (*Ximenia americana*).

Cancer Cell Line (H: Human, M: Mouse, R: Rat, Hamster: h)	Lectin	Toxicity	Proliferation Inhibition	Ref.
HT29 colon (H), MCF-7 breast (H)	ABL	-	+	[153]
HeLa (H), SW480 lymph node metastasis (H); SGC-7901, BGC-823	AAL	+ (M)	+	[35]
gastric cancer (H); MGC80-3 gastric adeno-carcinoma (H); HL-60				
leukemia (H); S-180 sarcoma (M)				
NCI-60 tumor cell line panel (H), LOX IMVI melanoma (H)	BfL	-	+	[46]
SK-MEL-28 melanoma (H), HT-144 melanoma (H), C32 melanoma	GSA-IA_4_	+	+	[54,154]
(H), LS174t, SW1116 colon cancer (H)				
A431 epidermoid carcinoma (H); HT29 colorectal carcinoma (H)	Jacalin, PNA	+	+	[155,156]
JAr choriocarcinoma (H); H3B hepato-carcinoma (H); B16				
melanoma (M)				
EAC Ehrlich ascites carcinoma; A549 lung carcinoma (H); CNE-1	MCL	+	+	[157,158,159]
CNE-2 nasopharyngeal carcinoma (H)				
BT20, BT549, MCF7, HS578T, HBL100, T47D breast cancer (H)	ML-I, -II, -III	+		[141,160]
SK-Hep-1, SK-Hep-3B hepatocarcinoma (H)				
HT-29 colon (H)	McL	+	+	[64]
G-361 melanoma (H); HepG2 hepatoma (H); SKGIIIa cervical	Ricin	+	+	[161,162]
carcinoma (H)				
Raji, Daudi lymphoma cell lines (H); JAr choriocarcinoma (H);	SBA	+	-	[155,163]
H3B hepato-carcinoma (H); B16 melanoma (M)				
HT-29 colon (H)	SRL	+	+	[164]
MCF7, MDA-MB231 breast carcinoma (H); U87-MG brain tumor (H)	Riproximin	+	-	[85]
HEp2 larynx (H); NCI-H460 lung (H); HT29 colon (H); PC3				
prostate (H); SKW3, K562, BV173 leukemia (H)				

**Table 3:** Mechanisms involved in the cytotoxic effects of lectins on cancer cells.

Lectin	Mechanism	Ref.
(*Abrus precatorius*) Abrin	Abrin (type II RIP) induced the caspase 3-dependent but caspase 8-independent apoptotic pathway, mitochondrial membrane potential damage and production of ROS in Jurkat cells.	[165]
(*Abrus precatorius*) *A. p*. lectin	Peptides from *A. p.* lectin induced drastic loss of mitochondrial membrane potential and increase in ROS, leading to symptoms of early apoptosis through a deregulation of Akt and activation of both JNK, MAPK, p53 and autophagy in HeLa cells.	[166]
(*Abrus precatorius*) Abrin P2	Abrin P2 suppressed the proliferation of colon HCT-8 cell line and provoked a cell cycle arrest at the S and G2/M phases. Abrin P2 inhibited cell proliferation via the down-regulation of cyclin B1 and the nuclear antigen Ki67, and the up-regulation of P21. The abrin P2-induced apoptosis was dose- and time-dependent.	[167]
(*Abrus precatorius*) agglutinin AGG	AGG administered to human breast xenografted athymic nude mice mediated anti-tumorigenic effects through induction of extrinsic apoptosis via Akt-dependent ROS generation, and inhibition of angiogenesis via inhibition of expression of the pro-angiogenic factor IGFBP2 in an AKT-dependent manner.	[168]
(*Agrocybe aegerita*) lectin AAL	AAL inhibited the growth of different tumor cell lines HeLa, SW480, SGC-7901, MGC80-3, BGC-823 and HL-60 and induced apoptosis in HeLa cells. It also displayed DNAse activity.	[35]
(*Arachis hypogaea*) peanut agglutinin PNA	PNA induced autophagy and apoptotic cell death in HeLa cells, associated to a concomitant increase in ROS.	[169]
(*Artocarpus integrifolia*) jacalin	Rounding of A431 (epidermoid carcinoma) and HT29 (colorectal carcinoma) cells due to the stress-induced phosphorylation of caveolin-1 and p38 and down-regulation of EGFr.	[155]
(*Bauhinia forficata*) lectin BfL	BfL inhibited the adhesion of breast cancer MCF7 cells on laminin, collagen I and fibronectin, decreased the α1, α6 and β1 integrin subunit expression and increased the α5 subunit expression. BfL caused necrosis of MCF7 cells with caspase-9 inhibition, DNA fragmentation and cell cycle arrest in the G2/M phase.	[170]
(*Glycine max*) soybean agglutinin SBL	SBL-mediated autophagy, apoptosis and DNA damage in HeLa cells depend on the generation of ROS. Pre-treatment of HeLa cells by the ROS scavenger *N*-acetylcysteine reduced both SBL-induced autophagy, apoptosis and DNA damage.	[171]
(*Momordica charantia*) lectin MCL	MCL induced apoptosis, DNA fragmentation, G1 phase arrest and mitochondrial injury in nasopharyngeal carcinoma NPC cells in vitro and in vivo, associated with regulation of p38 MAPK, NK and ERK phosphorylation and NO production. MCL increased cytochrome c release in the cytosol, activated caspase-3, -8 and -9 and enhanced production of PARP.	[157]
(*Momordica charantia*) lectin MCL	MCL treatment induced G2/M phase arrest, autophagy, DNA fragmentation, mitochondrial injury and apoptosis in HCC cells. Activation of caspase and MAPK pathway was involved in the MCL-induced apoptosis. Up-regulation of truncated Bid (tBid) was shown to occur during the MCL treatment.	[172]
(*Momordica charantia*) RIP MAP30	MAP30 from *Momordica charantia* promotes apoptosis in both Hep G2 cells (hepatocellular carcinoma) and Hep G2-bearing mice. The contribution of both caspase-8 regulated extrinsic and caspase-9 intrinsic caspase cascades was evidenced.	[173]
(*Momordica charantia*) α-momorcharin and MAP30	Both RIPs induced cell cycle arrest in S-phase, DNA fragmentation and apoptosis in A549 lung carcinoma cells. Inhibition of cell proliferation was dose- and time-dependent.	[158]
(*Sambucus nigra*) agglutinin SNA	SNA activates the signaling pathways of AKT and ERK1/2 in ovarian carcinoma cells. The mitochondrial outer membrane permeabilization resulted in ROS generation and cytochrome c release in the cytosol. The perturbed mitochondrial respiration resulted in the G2/M phase cell cycle arrest.	[174]
(*Sclerotium rolfsii*) lectin SRL	SRL caused dose-dependent inhibition of proliferation of MCF-7 and ZR-75 breast cancer cells via induction of cellular apoptosis. Inhibitors of caspase-3, -8 and -9 prevented the apoptosis to occur.	[164]
(*Viscum album*) Korean mistletoe lectin VCA	VCA elicited apoptosis in SK-Hep-1 p53-positive and Hep 3B p53-negative hepatocarcinoma cell lines by down-regulation of Bcl-2 and up-regulation of Bax functioning upstream of caspase-3. Down-regulation of telomerase activity occurred in both VCA-treated cells.	[141]
(*Viscum album*) Mistletoe lectin-1 ML-1	CM-1 induced apoptosis in colorectal cancer cells by down-regulating the miR-135a&b miRNAs expression. The expression of β-catenin was up-regulated.	[149]
(*Viscum album*) Recombinant aviscumine	The mechanism of aviscumin-mediated cell death on multiple cell types was solely induced by the toxic A-chain. The mechanism is independent from the death receptor Fas and independent of the activity of the anti-apoptotic transcription factor NFκB. Treatment with aviscumine inhibited growth in various metastases mouse models including C8 colon carcinoma, Lewis lung sarcoma, Renca renal sarcoma, etc.	[175]
(*Viscum album*) Korean mistletoe lectin VCA	Treatment of B16BL6 and B16F10 melanoma cells with VCA resulted in G0/G1 phase arrest and induced an increase in both early and late apoptosis. Both VCA and mistletoe extracts increased activated multiple caspases (caspase-1, 3, 4, 5, 6, 7, 8 and 9) and a decrease of procaspase 3 and 8.	[176]
(*Ricinus communis*) agglutinin RCA and ricin A-chain	Treatment of cancer cells in vitro by ricin and ricin A-chain activates caspase 3 and caspase 8, but not caspase 9. In vivo, cell death depends on the necrotic effect of the RIP.	[177]
(*Ricinus communis*) ricin	Ricin inhibited the proliferation of HeLa cells by inducing apoptosis, chromatin condensation and nuclear fragmentation.	[178]
(*Ricinus communis*) ricin and riproximin	Unfolding protein response UPR to endoplasmic reticulum stress was induced in both HCT116 and MDA-MB-231 cells. Apoptosis was induced by concentrations of RIPs-II at which the UPR-related genes are still translated.	[179]
(*Viscum album*) Korean mistletoe lectin-II	Lectin-II induced the activation of caspase-3, -8 and -9 of myeloleukemic U937 cells in a time- and dose-dependent manner.	[180]
(*Viscum album*) mistletoe lectin-II	Apoptotic cell death of U937 cells was induced by the generation of pro-oxidants mediating the JNK/SAPK activation, cytochrome c release, activation of caspase-9- and -3-like proteases, and PARP cleavage.	[181]
(*Viscum album*) Korean mistletoe lectin VCA	Induction of apoptosis in A253 cells through activation of caspase-3 and inhibition of telomerase activity through transcriptional down-regulation of hTERT. Inhibition of telomerase activity resulted from dephosphorylation of Akt.	[182]
(*Viscum album*) European mistletoe lectin-containing extracts	In vitro and ex vivo treatment of Ewing sarcoma cells by mistletoe extracts inhibited proliferation and induced a dose-dependent apoptosis via intrinsic and extrinsic apoptotic pathways, as evidenced by activation of both caspase-8 and caspase-9.	[183]
(*Viscum album*) European mistletoe lectin-containing extracts	Treatment of Ewing sarcoma cells by mistletoe extracts impacted both gene and protein expression. Cell response to oxidative stress induced the activation of the MAPK signaling pathway.	[184]
(*Ximenia americana*) riproximin	Riproximin induced cytotoxic effects on breast cancer cell lines MDA-MB-231 and MCF-7. Riproximin treatment caused arrest in S phase and nuclear fragmentation of the cell, induced cytokine IL24/MDA-7 and ER-stress-related GADD genes. An inhibition of the genes involved in migration of colony was observed.	[185]
